# Electrostatic and Environmental Control of the Trion Fine Structure in Transition Metal Dichalcogenide Monolayers

**DOI:** 10.3390/nano12213728

**Published:** 2022-10-24

**Authors:** Yaroslav V. Zhumagulov, Alexei Vagov, Dmitry R. Gulevich, Vasili Perebeinos

**Affiliations:** 1 Faculty of Physics, University of Regensburg, 93040 Regensburg, Germany; 2 Faculty of Physics, National Research University Higher School of Economics, 101000 Moscow, Russia; 3 ITMO University, 197101 St. Petersburg, Russia; 4 Department of Electrical Engineering, University at Buffalo, The State University of New York, Buffalo, NY 14260, USA

**Keywords:** excitons, trions, transition metal dichalcogenides

## Abstract

Charged excitons or trions are essential for optical spectra in low-dimensional doped monolayers (ML) of transitional metal dichalcogenides (TMDC). Using a direct diagonalization of the three-body Hamiltonian, we calculate the low-lying trion states in four types of TMDC MLs as a function of doping and dielectric environment. We show that the fine structure of the trion is the result of the interplay between the spin-valley fine structure of the single-particle bands and the exchange interaction. We demonstrate that by variations of the doping and dielectric environment, the fine structure of the trion energy can be tuned, leading to anticrossing of the bright and dark states, with substantial implications for the optical spectra of the TMDC ML.

## 1. Introduction

The 2D geometry of TMDC ML significantly enhances the Coulomb interaction, leading to a much higher exciton binding energy [1,2,3,4,5,6] compared to bulk semiconductors [7,8,9]. 2D confinement also facilitates other many-body states, including three-particle trions [10,11,12,13,14,15,16,17,18,19] and four-particle biexcitons [20,21,22,23,24,25,26] with high binding energies. Experiments and theoretical calculations demonstrated that the lowest-energy optical excitations in a doped TMDC ML are often associated with trions [10,11,12,13,14,15,16,17,18,19]. A sophisticated band structure with two direct band gaps at the *K* and $K^{\prime }=-K$ points of the Brillouin zone and the presence of the spin valley locking effect [27,28,29,30] allow various trionic states to be realized in the TMDC ML [31,32,33,34,35,36,37,38,39,40,41,42,43,44,45]. Commonly, trions are classified by spin and valley quantum numbers, and they can be dark or bright depending on the combination of the quantum numbers. The trionic fine structure determines the optical absorption edge and the photoluminescence spectra. In particular, the photoluminescence spectra strongly depend on whether the ground trion state is optically bright or dark.

The low energy trion spectra in TMDC MLs are crucially dependent on multiple factors, such as spin-orbit coupling, direct and exchange Coulomb interactions, and carrier doping level. The dark- and bright-state energies determine the spectral properties of TMDC, particularly the temperature behavior of the photoluminescence spectra [40,46,47,48]. The internal structure of the trion states makes them optically dark or bright, depending on the valley and spin configuration. Because of the small energy differences, these states are difficult to distinguish experimentally, especially at elevated temperatures and in samples with a significant number of defects. For the same reasons, it is not easy to accurately calculate the trion states’ relative positions. For example, MoS2 ML theories [36,39,49] predict different types of the lowest energy trion states. The experiments observe bright trions in the photoluminesce spectra, and positions of the dark states can only be inferred from an additional set of measurements, such as temperature dependence of the photoluminescence intensity [32] or excited state dynamics [50].

This work presents a comprehensive theoretical analysis of the three-particle states in TMDC MLs. We find that the low-energy fine structure of trions depends not only on the material-specific band structure but also on the doping and dielectric environment. The calculated trion states are mainly controlled by the interaction of spin-orbit splitting and exchange interactions, which depend on doping and the environment. The most nontrivial situation arises when the spin-orbit splitting is small and negative, as in MoS2 ML. In this case, we find that the low-lying trion states are close to degeneracy, allowing the possibility of switching the relative positions of the dark and bright states by varying the external gate voltage in a field-effect transistor setup on different dielectric substrates. However, because the predicted tunability range of energies is only a few meV, the design of low-temperature experiments using homogeneous samples with low defect density is needed to observe the anticrossing phenomena. While bright trions reveal themselves in the photoluminesce spectra, additional measurements are needed, such as temperature dependence of the photoluminesce, excited states dynamics, or non-linear spectroscopy to probe the dark states. Thin oxide dielectrics can help to establish higher gate capacitance for a broader range of Fermi-level tunability.

## 2. Materials and Methods: Three-Particle States in TMDC Monolayers

The calculation of trion states is performed by a direct diagonalization of the three-body Hamiltonian obtained by spanning the many-body model into states with two electrons and a hole (we consider negatively charged trions) $|c_{1}c_{2}v\rangle =a_{c_{1}}^{†}a_{c_{2}}^{†}a_{v}|0\rangle$, where $c_{1,2}$ and *v* are single-particle electron and hole states. The ground state $|0\rangle$ has a filled valence band and an empty conduction band. The Hamiltonian reads as follows.

(1) $$\begin{array}{cc} H_{\;\;}= & H_{0}+H_{cc}+H_{cv},\\ H_{0}= & +\left(\varepsilon_{c_{1}}+\varepsilon_{c_{2}}-\varepsilon_{v}\right)\delta_{c_{1}}^{c_{1}^{\prime }}\delta_{c_{2}}^{c_{2}^{\prime }}\delta_{v}^{v^{\prime }},\\ H_{cc}= & +\left(W_{c_{1}c_{2}}^{c_{1}^{\prime }c_{2}^{\prime }}-W_{c_{1}c_{2}}^{c_{2}^{\prime }c_{1}^{\prime }}\right)\delta_{v}^{v^{\prime }},\\ H_{cv}= & -\left(W_{v^{\prime }c_{1}}^{vc_{1}^{\prime }}-V_{v^{\prime }c_{1}}^{c_{1}^{\prime }v}\right)\delta_{c_{2}}^{c_{2}^{\prime }}-\left(W_{v^{\prime }c_{2}}^{vc_{2}^{\prime }}-V_{v^{\prime }c_{2}}^{c_{2}^{\prime }v}\right)\delta_{c_{1}}^{c_{1}^{\prime }}\\ & +\left(W_{v^{\prime }c_{1}}^{vc_{2}^{\prime }}-V_{v^{\prime }c_{1}}^{c_{2}^{\prime }v}\right)\delta_{c_{2}}^{c_{1}^{\prime }}+\left(W_{v^{\prime }c_{2}}^{vc_{1}^{\prime }}-V_{v^{\prime }c_{2}}^{c_{1}^{\prime }v}\right)\delta_{c_{1}}^{c_{2}^{\prime }}, \end{array}$$

where $\varepsilon_{c,v}$ are single-particle energies, *W* and *V* are the screened and bare Coulomb potentials. The latter is given by $V_{cd}^{ab}=V\left(k_{a}-k_{c}\right)\langle u_{c}|u_{a}\rangle \langle u_{d}|u_{b}\rangle$ with $\langle u_{c}|u_{a}\rangle$ and the overlap of the Bloch states of a single particle and $V\left(q\right)=2\pi e^{2}/q$. For the intravalley screened potential, we follow Reference [51] and substitute V(q) in this expression by the Rytova-Keldysh W(q) potential [52,53,54,55]

(2) $$\begin{array}{c} W\left(q\right)=V\left(q\right)\left\{\begin{array}{cc} \varepsilon_{env}^{-1}{\left(1+r_{0}q\right)}^{-1} & q-\mathrm{intravalley}\\ \varepsilon_{bulk}^{-1} & q-\mathrm{interavalley} \end{array}\right., \end{array}$$

where “intravalley” stands for the states within the same valley and “intervalley” stands for states in different valleys. For the encapsulating material, the effective dielectric constant $\varepsilon_{env}=\left(\varepsilon_{2}+\varepsilon_{1}\right)/2$ is the average of dielectric constants on both sides of the ML, and the screening length is $r_{0}=\varepsilon_{bulk}d/\left(2\varepsilon_{env}\right)$ with *d* being the width of the ML [51,56]. Notice that the finite-intervalley dielectric screening induces the intervalley interaction between pairs of trion states. It was not observed in previous works where only the Keldysh potential was used [40,57].

For the single-particle states, we assume the massive k·p Dirac model [58]

(3) $$\begin{array}{cc} H_{0} & =v_{F}{\hat{s}}_{0}⊗\left(\tau k_{x}{\hat{\sigma }}_{x}+k_{y}{\hat{\sigma }}_{y}\right)+\frac{\Delta }{2}{\hat{s}}_{0}⊗{\hat{\sigma }}_{z}\\ \; & +\frac{1}{2}\tau {\hat{s}}_{z}⊗\left[\lambda_{c}\left({\hat{\sigma }}_{0}+{\hat{\sigma }}_{z}\right)+\lambda_{v}\left({\hat{\sigma }}_{0}-{\hat{\sigma }}_{z}\right)\right], \end{array}$$

where ${\hat{\sigma }}_{i}$ are the Pauli matrices in the band subspace, ${\hat{s}}_{z}$ is the Pauli matrix in the spin subspace, ${\hat{\sigma }}_{0}$ and ${\hat{s}}_{0}$ are unity matrices, τ=±1 is the valley index for *K* and $K^{\prime }=-K$, $v_{F}$ is the effective Fermi velocity, and Δ is the bandgap. The last contribution to Equation (3) describes the Zeeman spin-orbit coupling with constants $\lambda_{c,v}$. The parameters Δ and $\lambda_{c,v}$ are obtained by fitting the ab initio band structure calculations using the DFT/GW approaches [59,60,61]. The gap Δ depends on the encapsulating materials and needs a correction, for example, using the scissor operator approach, when quantitatively accurate results are required [56]. It is known that the dielectric environment does not change the effective mass of the single-particle states [62]. To reflect this fact, the effective Fermi velocity is chosen as $v_{F}=\sqrt{\Delta /2m}$. Pauli blocking accounts for doping in a finite-grid mesh of the reciprocal space [40,57,63]. The dipole matrix elements needed to calculate the oscillator strength are obtained from the trion wavefunctions. Further details of the calculations can be found in Appendix A and Appendix B, and the model parameters, extracted from the first-principles calculations for TMDC ML, are summarized in Table 1.

## 3. Results

**Figure 1:**
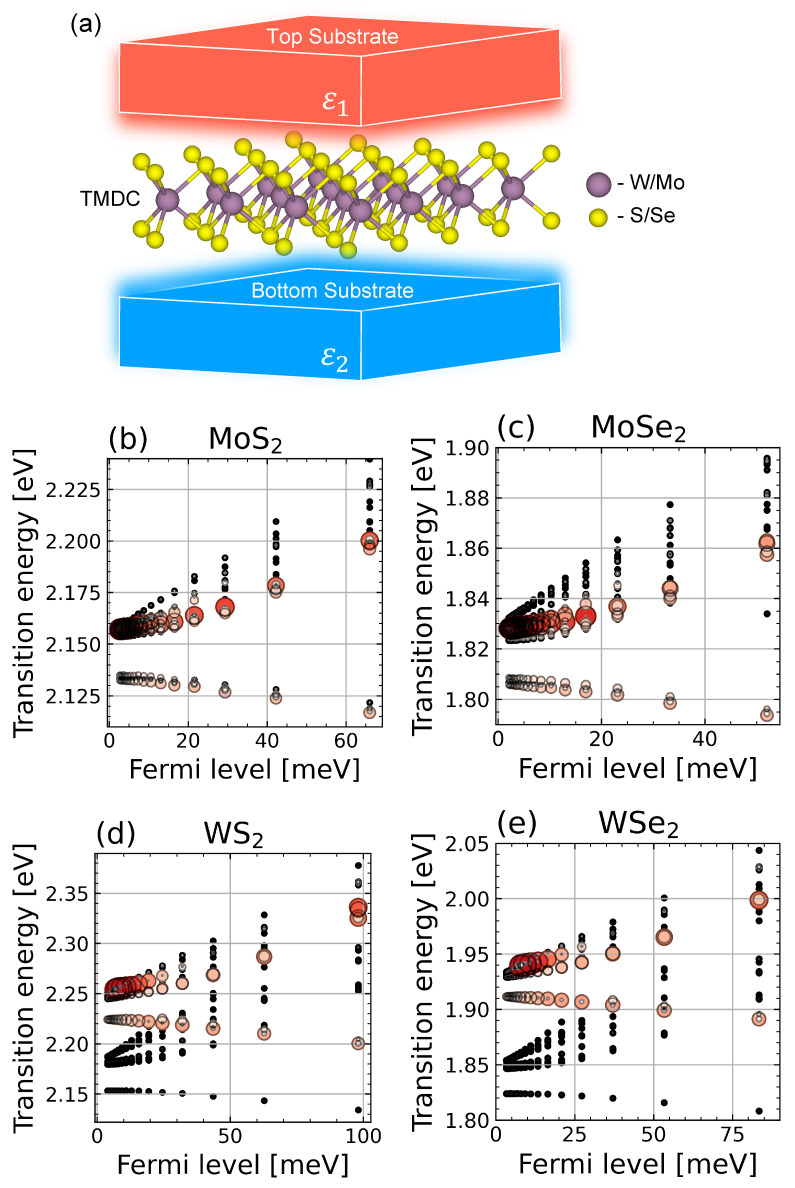
(**a**) Schematics of the TMDC monolayer placed between two dielectrics that define environmental screening $\varepsilon_{env}=\left(\varepsilon_{1}+\varepsilon_{2}\right)/2$. (**b**–**e**) Energy diagram of the transition energies of the three-particle states calculated in freestanding ($\varepsilon_{env}=1$) ML of MoS2 (**b**), MoSe2 (**c**), WS2 (**d**), and WSe2 (**e**) as a function of the Fermi level. Circle centers denote optical transition energies of trions. Colors and widths indicate their oscillator strengths (red) for bright states and (black) for dark ones. “Trion” and “exciton” denote trion and exciton states, depending on whether the second electron in the three-particle wavefunction is bound or not.

Results of the calculations for three-particle states in MoS2, MoSe2, WS2, and WSe2 MLs is given in Figure 1, where we plot optical transition energies of trions (circle positions) and their oscillator strengths (circle size and color) as a function of the electron doping (the relative Fermi energy of the doping electrons). These are obtained by directly solving an effective three-particle Hamiltonian.

We distinguish two qualitatively different three-particle states: “trions”, where both electrons are tightly bound, and “excitons”, where one of the electrons is almost free, so that the state is close to being a two-particle exciton weakly coupled to a single electron [40,57,63]. The difference between excitons and trions is also clearly manifested in the doping dependence in Figure 1. It shows that the excitons’ energy increases at larger doping, whereas, for tightly bound trions, the doping dependence is much weaker.

A tightly bound trion is the lowest-energy state. In WS2 and WSe2, this state is dark for all doping values, and in MoSe2, it is bright. The first optical transition due to the higher energy bright trion state is separated from the lowest one by a relatively large gap, ΔE≃20 meV, consistent with the GW-BSE approach for this energy difference [50].

On the contrary, MoS2 has four different but energetically closely spaced low-energy trion states, denoted T${}_{1}$, T${}_{2}$, T${}_{3}$, and T${}_{4}$, which give rise to transition energies within ΔE≃2 meV. Apart from the near-degeneracy, those trion states reveal another exciting feature: doping can change the brightness of the lowest energy state, which is dark at low doping values and brightens when doping increases. Doping also significantly changes the energy splitting of these states.

The fine structure and near degeneracy of these trion states in MoS2 ML is closely related to the spin-orbit coupling in the single-particle Hamiltonian, which appears in TMDCs due to the valley Zeeman splitting of the conduction band. At the same time, according to ab initio calculations, the Rashba coupling is minimal [58,59,61]. The calculations show that the Zeeman valley splitting is large in W-based materials [58,59,61]. Its value is reflected in the large gap between the lowest and the first excited bright trion states (Figure 1c,d). The splitting is also notable in the MoSe2 structure (Figure 1b). However, in the latter case, the splitting has a negative value, leading to a bright state of the lowest energy in MoSe2, unlike the W-based materials. In contrast, in MoS2, the valley Zeeman splitting is small, which is the main reason why the energies of the four lowest energies of the trion states are very similar.

## 4. Discussion

### 4.1. Symmetry and Internal Structure of Trions

**Figure 2:**
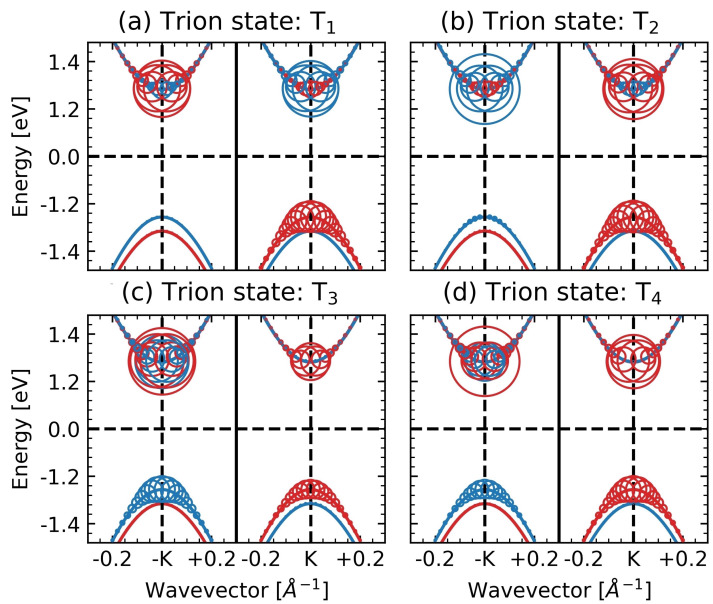
Contributions of Dirac single-particle band states in the vicinity of the *K* and −K points to trions (panels (**a**–**d**)). A circle position points to the single-particle state and its radius gives weight to the exciton state; colors mark the spin projection $s^{z}$. The states T${}_{1}$ and T${}_{2}$ in panels (**a**,**b**) have $\tau s^{z}=1/2$, and the states T${}_{3}$ and T${}_{4}$ in panels (**c**) and (**d**) have $\tau s^{z}=-1/2$. Results are shown for $E_{F}=2.9$ meV.

The internal structure of trion states, particularly the weights of the contributions of the single-particle states, is the key to understanding their optical properties. Single-particle contributions (single-particle density matrix) for the T${}_{1}$–T${}_{4}$ trions, calculated for a freestanding MoS2 ML with the doping level of $E_{F}=2.9$ meV, are illustrated in Figure 2. The figure shows single-particle bands for electrons (positive energies) and holes (negative energies) in the vicinity of the *K* and −K valley points in the Brillouin zone. The blue and red denote the spin $s^{z}=\pm 1/2$ state. The center of a circle indicates the contribution of the single-particle state energy, whereas its radius indicates the contribution weight of this state to the trion wavefunction. Strong intervalley mixing by the Coulomb interaction creates a superposition of many single-particle states [42,65,66] so that a simple representation of trions as a product of a few states is generally impossible.

Note that trion states are classified by the valley quantum number $\tau =\tau_{c_{1}}+\tau_{c_{2}}-\tau_{v}$ and by the spin quantum number $s^{z}=s_{c_{1}}^{z}+s_{c_{2}}^{z}-s_{v}^{z}$ given as sums of the corresponding single particle numbers. We consider only states with $|\tau s^{z}|=1/2$, which is a necessary (but not sufficient) condition for a state to be bright. The fine structure of $|\tau s^{z}|=3/2$ states is discussed in Appendix C. If $|\tau s^{z}|\neq 1/2$, then the state does not have optically active electron-hole pairs contributing to the three-particle trion wavefunction. The T${}_{1}$–T${}_{4}$ states are split into qualitatively different pair states: T${}_{1}$ and T${}_{2}$ with $\tau s^{z}=1/2$ and T${}_{3}$ and T${}_{4}$ with $\tau s^{z}=-1/2$.

The pair of trions T${}_{3}$ and T${}_{4}$ is a well-known singlet-triplet pair observed in earlier work [32,35,44,45,50,63,67,68,69,70], experimental and theoretical. In the T${}_{1}$ and T${}_{2}$ trion states, a hole is provided by a single valley (−K), while both valleys contribute electrons of different spin. In the vanishing doping limit, T${}_{1}$ is dark and T${}_{2}$ is bright because the valley electrons in these states have opposite spins [see Figure 2]. However, both the T${}_{1}$ and T${}_{2}$ states brighten as doping increases. At the same time, the T${}_{3}$ and T${}_{4}$ states comprise holes of opposite spins. In the limit of zero doping, T${}_{4}$ is an optically parity forbidden state, while T${}_{1}$ is an optically spin forbidden state. Notice that in addition to the T${}_{1}$–T${}_{4}$ trions, the system has four equivalent states, related by the symmetry transformation K↔−K.

### 4.2. Manipulating the Lowest Trion States

**Figure 3:**
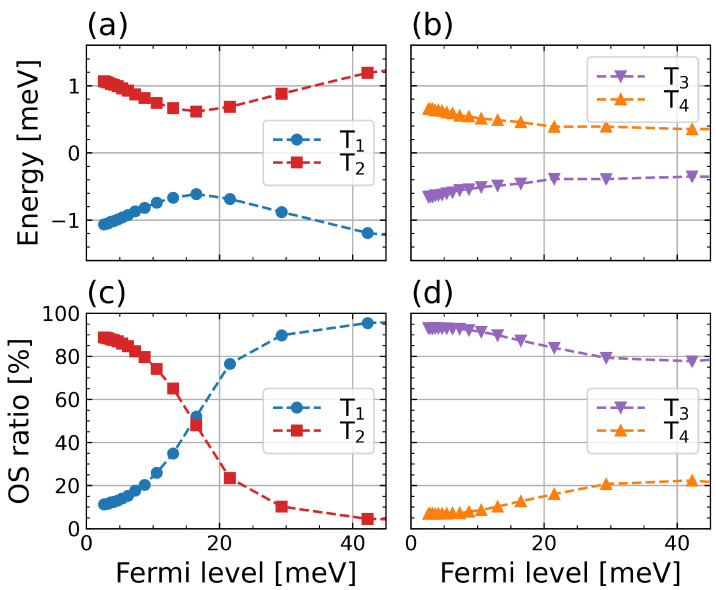
Doping dependence of relative transition energies of trion states T${}_{1}$ and T${}_{2}$ (**a**) and T${}_{3}$ and T${}_{4}$ (**b**) and relative oscillator strength of trion states T${}_{1}$ and T${}_{2}$ (**c**) and T${}_{3}$ and T${}_{4}$ (**d**) calculated for a free-standing MoS2 ML. The transition energies in (**a**,**b**) are shown with respect to their average at each doping level.

The near degeneracy of the trion states in MoS2 makes their energies and OS very sensitive to the parameters of the system, allowing a way to efficiently control the brightness of the lowest energy trion. In Figure 3a,b, we demonstrate anticrossing between the pairs of T${}_{1}$–T${}_{2}$ and T${}_{3}$–T${}_{4}$ states by subtracting the average values from their transition energies. One can see that the T${}_{1}$ and T${}_{3}$ states are the lowest energy states in the respective pairs, and the T${}_{1}$ and T${}_{4}$ states are dark at the zero doping limit, as shown in Figure 3c,d. The increase in doping brightens these initially dark states. Figure 3c shows that the oscillator strength of the T${}_{1}$ state increases, whereas, for the T${}_{2}$ state, it drops, such that the OSs of the two states become equal at $E_{F}≃16$ meV. This interchange in brightness is accompanied by an energy change with the doping. The resulting behavior looks similar to a standard anti-crossing pattern, as seen in Figure 3a,b. The same brightness interchange and anticrossing is also observed for the other pair of states, T${}_{2}$ and T${}_{4}$. However, in this case, the anticrossing point is shifted to higher values of the doping level, that is, $E_{F}≃50$ meV.

**Figure 4:**
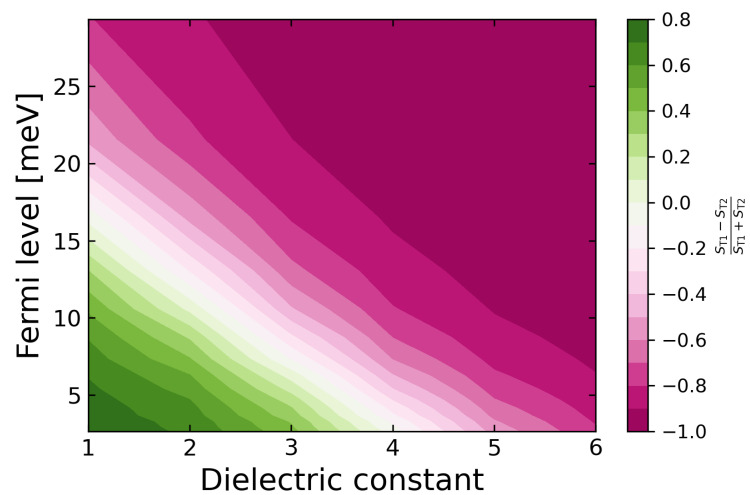
Dependence of the oscillator strength $S_{T_{1},T_{2}}$ of the T${}_{1}$ and T${}_{2}$ trion states on doping. The color scale shows the ratio $\left(S_{T_{1}}-S_{T_{2}}\right)/\left(S_{T_{1}}+S_{T_{2}}\right)$.

The fine structure of these trions can also be controlled by choosing the dielectric environment that embeds the ML. Figure 4 shows the color density plot for the oscillator strength ratio of the T${}_{1}$ and T${}_{2}$ states as a function of both the Fermi level $E_{F}$ and the dielectric constant $\varepsilon_{env}$. The white dashed line in Figure 4 represents the anticrossing position corresponding to the doping level, where the oscillator strengths of both peaks are equal. One can see that, at vanishing doping, the lowest energy trion state is dark when $\varepsilon_{env}<4$ and bright when $\varepsilon_{env}>4$. This strong influence of the environment on the fine structure of the lowest-energy trion can, in principle, explain a discrepancy observed in different calculations and measurements with different conclusions on the brightness of the lowest-energy trion state in MoS2 [32,36,39,49,50]. Our calculations demonstrate that this discrepancy is due to the near-degeneracy, strong dependence on the environment, and influence of doping on the fine structure of the lowest-energy trion states. At the same time, we find that the environment has only a marginal effect on the states T${}_{3}$ and T${}_{4}$.

### 4.3. The Role of Spin-Orbit and Exchange Interactions

The fine structure of the trion states in MoS2 is defined by the small spin-orbit coupling and its interaction with the Coulomb exchange interaction, which determines the anticrossing mechanism in the trion states. To demonstrate this, we first note that despite a large admixture of many three-particle states, the pairs of states T${}_{1}$–T${}_{2}$ and T${}_{3}$–T${}_{4}$ are only weakly coupled to each other and other trion states of the higher energy. This fact is intuitive because these pairs have different values of $\tau s_{z}=\pm 1$ and therefore differ by their symmetry, which is also discussed in Reference [35]. At the same time, these states are practically uncoupled from the higher-energy states separated by a large gap [see Figure 1].

The interaction between trions is then pairwise and can then be modeled using a simple two-level effective Hamiltonian:

(4) $$H=\left[\begin{array}{cc} \epsilon_{1} & g\\ g & \epsilon_{2} \end{array}\right].$$

Here, diagonal elements comprise single- and many-body components $\epsilon_{i}=\langle i|H|i\rangle =\epsilon_{i}^{\left(0\right)}+\epsilon_{i}^{\left(1\right)}$, and their difference $\Delta =\epsilon_{1}-\epsilon_{2}$ controls anticrossing.

For the pair T${}_{1}$–T${}_{2}$, a two-level basis is represented by the states $\left|1\rangle =\right|K_{↓},-K_{↑},-K_{↓}\rangle$ (dark) and $\left|2\rangle =\right|K_{↑},-K_{↓},-K_{↓}\rangle$ (bright). The single particle contribution to the energy difference $\Delta^{\left(0\right)}=2\lambda_{c}$ follows from the spin-orbit splitting of the conduction band, whereas the many-body contribution $\Delta^{\left(1\right)}≃V_{\mathrm{exc}}^{cv}\left(K=0\right)>0$ is defined mainly by the intravalley electron-hole exchange interaction. The off-diagonal coupling elements $g=W_{\mathrm{exc}}^{cc}\left(K\right)<0$ are defined by the intervalley electron-electron exchange interaction, which is a few meV [66]. The anticrossing condition for this pair $\Delta_{T{}_{1}–T{}_{2}}≃2\lambda_{c}+V_{\mathrm{exc}}^{cv}\left(K=0\right)=0$ can be satisfied when $\lambda_{c}$ is relatively small and negative, and this indeed occurs in MoS2.

At the same time, the basis for the T${}_{3}$ and T${}_{4}$ states is represented by $\left|1\rangle =\right|K_{↓},K_{↑},K_{↑}\rangle$ (bright) and $\left|2\rangle =\right|K_{↓},-K_{↓},-K_{↓}\rangle$ (bright). The energy difference $\Delta_{T{}_{3}–T{}_{4}}=\epsilon_{1}-\epsilon_{2}=\epsilon_{1}^{\left(1\right)}=W_{\mathrm{dir}}^{cc}\left(K=0\right)>0$ between the diagonal elements is mainly defined by the direct electron-electron intravalley interaction, which is the many-body component of the diagonal energy of the state |1〉. The single-particle contributions in both states are the same and yield zero difference. The off-diagonal coupling in this case $g=V_{\mathrm{exc}}^{cv}\left(K\right)>0$ is obtained by hopping electron-hole intervalley pairs due to the intervalley electron-hole exchange interaction [71,72,73]. In the zero doping level limit, the interaction between the T${}_{3}$ and T${}_{4}$ trions leads to the T${}_{4}$ state becoming optically forbidden. In contrast to the T${}_{1}$–T${}_{2}$ pair, the T${}_{3}$–T${}_{4}$ trion pair has strong off-diagonal coupling in all TMDC monolayers, whereas the diagonal energy difference $\Delta_{T{}_{3}–T{}_{4}}$ does not depend on $\lambda_{c}$ and is practically identical for all TMDCs. In all TMDC monolayers, the pair T${}_{1}$–T${}_{2}$ yields the lowest energy ground state. This implies that the ground state of the trion can be efficiently controlled by the doping and dielectric environment only in the MoS2 monolayers.

## 5. Conclusions

The analysis of three-particle states in TMDC MLs, based on a direct diagonalization of the corresponding Hamiltonian, classifies the low-energy trion states’ fine structure and spectral properties of doped TMDCs. In particular, we demonstrate the bright-dark interchange of the lowest-energy trions as a function of doping and the dielectric environment. The calculations reveal that the energies and oscillator strengths of the lowest-energy trion states are defined mainly by the spin-orbit coupling and its interplay with the exchange interaction. When those quantities are similar, a material can exhibit a near-degeneracy of its four lowest-energy trion states, making them sensitive to the system parameters. This sensitivity opens the possibility of manipulating the position and brightness of the lowest-energy trion and thus the low-energy spectrum, simply by changing the electrostatic doping and choosing the dielectric environment. In particular, this situation can easily be realized in MoS2, where the sensitivity of the fine structure of the trion explains the origin of the controversy about whether the lowest trion energy is bright or dark. On the basis of the interplay between the fine-structure splitting and many-body effects, the controlling mechanism is generic and applies to other similar structures. Our results explain existing experimental observations of trion states in 2D multivalley materials and open new perspectives for their optoelectronic applications.

## Figures and Tables

**Table 1:** Model parameters for TMDC MLs: lattice constant *a*, effective mass *m* in units of free electron mass $m_{e}$, and spin-orbit couplings $\lambda_{c,v}$ are taken from Reference [61]. The layer thickness *d* and bulk dielectric constant $\varepsilon_{bulk}$ are from Reference [64], and bandgap $\Delta_{0}$ is from Reference [60] (see model (3)).

	*a* [Å]	*d* [Å]	$\varepsilon_{\mathrm{bulk}}$	$\Delta_{0}$ [eV]	$m/m_{e}$	$\lambda_{c}$ [meV]	$\lambda_{v}$ [meV]
MoS2	3.185	6.12	16.3	2.087	0.520	−1.41	74.60
MoSe2	3.319	6.54	17.9	1.817	0.608	−10.45	93.25
WS2	3.180	6.14	14.6	2.250	0.351	15.72	213.46
WSe2	3.319	6.52	16.0	1.979	0.379	19.85	233.07

**Figure A1:**
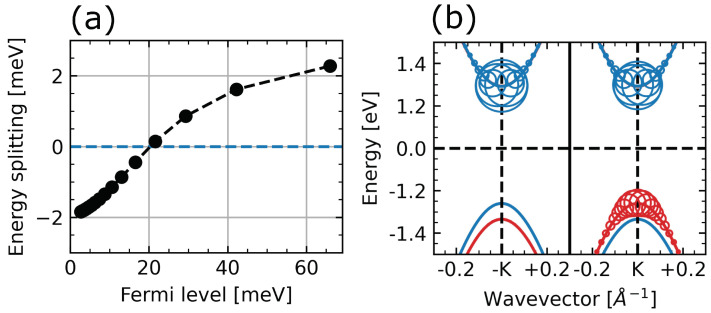
(**a**) Trion energy splitting between the lowest $s^{z}\tau =\pm 3/2$ state and the lowest $s^{z}\tau =\pm 1/2$ states of the freestanding of MoS2 monolayer. For a wide range of doping, the fully dark $s^{z}\tau =\pm 3/2$ state will be the lowest trion state of the freestanding of MoS2 monolayer. (**b**) Contributions of the Dirac single-particle band states in the vicinity of the *K* and −K points to the lowest $s^{z}\tau =\pm 3/2$ trion state.

## Data Availability

The data that support the findings of this study are available from the corresponding authors upon reasonable request.

## Abbreviations

The following abbreviations are used in this manuscript:

ML	monolayers
TMDC	transitional metal dichalcogenides
2D	two-dimensional
DFT	density functional theory
GW	GW approximation

## Appendix A. Computational Details: Single-Particle States

Trion states in a doped monolayer TDMC are calculated by solving an eigenvalue problem for the Hamiltonian for trion states constructed using a basis set of single-particle states in the valence and conduction bands. The doping influence is taken into account by connecting the discretization of the Brillouin zone with the doping-induced Pauli blocking [57]. We used the observation that the lowest energy trion states in TMDC monolayers are created from the single-particle states in the vicinity of points *K* and $K^{\prime }=-K$ of the Brillouin zone [36,57]. These states are well described by the massive Dirac Hamiltonian model in Equation (3).

We consider that the monolayer is embedded in a dielectric environment, with dielectric constants $\varepsilon_{1,2}$ for material on both sides of the monolayer. The environment affects the energy gap Δ, which can be expressed using an analytical formula [56]

(A1) $$\begin{array}{cc} \Delta & =\Delta_{0}+\frac{e^{2}}{2\varepsilon d}\left[\frac{L_{2}+L_{1}}{\sqrt{L_{2}L_{1}}}\tanh^{-1}\left(\sqrt{L_{2}L_{1}}\right)\right.\left.-\ln(1-L_{2}L_{1})\right],\;L_{i}=\frac{\varepsilon -\varepsilon_{i}}{\varepsilon +\varepsilon_{i}}, \end{array}$$

where ε is the dielectric constant of the bulk TMDC, *d* is the thickness of the monolayer and $\Delta_{0}$ are the bare values of the gap.

## Appendix B. Trion States

Quantum states *T* of negatively charged trions (two electrons and one hole) are obtained by solving the three-body eigenvalue problem derived by spanning the full many-body Hamiltonian onto the space of trion states constructed as a linear superposition

(A2) $$\begin{array}{c} |T\rangle =\sum_{c_{1},c_{2},v}A_{c_{1}c_{2}v}^{T}|c_{1}c_{2}v\rangle ,\;|c_{1}c_{2}v\rangle =a_{c_{1}}^{†}a_{c_{2}}^{†}a_{v}^{†}|0\rangle , \end{array}$$

where $c_{1,2}$ denote electron states in the conduction band, *v* are hole states in the valence band, and the ground state $|0\rangle$ has a filled valence band and an empty conduction band. Double counting is avoided by imposing the restriction $c_{1}<c_{2}$. The corresponding three-particle wavefunction is constructed from the single-particle functions $\phi_{c,v}\left(x\right)$ as

(A3) $$\begin{array}{cc} \Psi^{T}( & x_{1},x_{2},x_{3})=\frac{1}{\sqrt{2}}\sum_{c_{1},c_{2},v}A_{c_{1}c_{2}v}^{T}\phi_{v}^{*}\left(x_{3}\right)\times \left[\phi_{c_{1}}\left(x_{1}\right)\phi_{c_{2}}\left(x_{2}\right)-\phi_{c_{2}}\left(x_{1}\right)\phi_{c_{1}}\left(x_{2}\right)\right]. \end{array}$$

Both the electron and hole parts of the trion state are antisymmetric, which is reflected in the direct- and exchange-interaction potentials between electrons and holes. The representation of the wave function does not require explicit antisymmetrization between holes and electrons [74], which follows from a direct calculation of the wave function of excitons [75].

Coefficients $A_{c_{1}c_{2}v}^{T}$ in Equation (A2) are found by solving the matrix eigenvalue problem.

(A4) $$\begin{array}{c} \sum_{c_{1}^{\prime }c_{2}^{\prime }v^{\prime }}H_{c_{1}c_{2}v}^{c_{1}^{\prime }c_{2}^{\prime }v^{\prime }}A_{c_{1}^{\prime }c_{2}^{\prime }v^{\prime }}^{T}=E_{t}A_{c_{1}c_{2}v}^{T}. \end{array}$$

The Hamiltonian matrix $H=H_{0}+H_{cc}+H_{cv}$ comprises three contributions:

$$\begin{array}{cc} \; & H_{0}=\left(\varepsilon_{c_{1}}+\varepsilon_{c_{2}}-\varepsilon_{v}\right)\delta_{c_{1}c_{1}^{\prime }}\delta_{c_{2}c_{2}^{\prime }}\delta_{vv^{\prime }},\\ \; & H_{cc}=\left(W_{c_{1}c_{2}}^{c_{1}^{\prime }c_{2}^{\prime }}-W_{c_{1}c_{2}}^{c_{2}^{\prime }c_{1}^{\prime }}\right)\delta_{vv^{\prime }},\\ \; & H_{cv}=-\left(W_{v^{\prime }c_{1}}^{vc_{1}^{\prime }}-V_{v^{\prime }c_{1}}^{c_{1}^{\prime }v}\right)\delta_{c_{2}c_{2}^{\prime }}-\left(W_{v^{\prime }c_{2}}^{vc_{2}^{\prime }}-V_{v^{\prime }c_{2}}^{c_{2}^{\prime }v}\right)\delta_{c_{1}c_{1}^{\prime }}+\left(W_{v^{\prime }c_{1}}^{vc_{2}^{\prime }}-V_{v^{\prime }c_{1}}^{c_{2}^{\prime }v}\right)\delta_{c_{2}c_{1}^{\prime }}+\left(W_{v^{\prime }c_{2}}^{vc_{1}^{\prime }}-V_{v^{\prime }c_{2}}^{c_{1}^{\prime }v}\right)\delta_{c_{1}c_{2}^{\prime }}, \end{array}$$

where $\varepsilon_{c,v}$ denote the energy of the single-particle particle states, *W* and *V* are the screened and bare Coulomb matrix elements, respectively. This approach is a direct extension of the Tamm-Dancoff approximation for two-particle excitons onto three-particle trions. Matrix elements for the bare Coulomb interaction are given by

(A5) $$V_{cd}^{ab}=V\left(k_{a}-k_{c}\right)\langle u_{c}|u_{a}\rangle \langle u_{d}|u_{b}\rangle ,$$

where $V\left(q\right)=2\pi e^{2}/q$ is the Fourier component of the bare Coulomb potential and $\langle u_{c}|u_{a}\rangle$ is the overlap of the single-particle Bloch states *c* and *a*. The screened potential has a similar form to Equation (A5), however, instead of the bare Coulomb potential V(q) one uses the standard Rytova-Keldysh potential [52,53,54] given in Equation (2). Note that the screening depends on the geometry of the device. In particular, it is modified when the vacuum spacing appears between the monolayer and the dielectric environment [55]. However, in this work, this effect is neglected.

Symmetries of the three-particle Hamiltonian give rise to several conserved quantities, which helps to reduce the numerical efforts. These include the total trion momentum $k=k_{c_{1}}+k_{c_{2}}-k_{v}$ and *z* axis spin projection $s^{z}=s_{c_{1}}^{z}+s_{c_{2}}^{z}-s_{v}^{z}$. Furthermore, the analysis is restricted to trions with total spin 1/2, since only such states are optically active. Therefore, we neglect the states with spin 3/2 in the main text and consider them in the following.

The trion states are classified by the *z* component of the electron spin, $s_{c_{1}}^{z}+s_{c_{2}}^{z}=1$ and 0 [35,39,63,76,77], and distinguish between the intra- and inter-valley trion states by the quantities $\tau_{c_{1}}+\tau_{c_{2}}=2$ and 0, respectively [39,63]. The trion states can also be classified by the product $s_{z}\tau$ of the total spin projection $s^{z}$ and the total valley index $\tau =\tau_{c_{1}}+\tau_{c_{2}}-\tau_{v}$. A necessary (but not sufficient) condition for a state to be bright is $s^{z}\tau =\pm 1/2$.

Finally, the influence of electron doping is taken into account by using the approach described in Ref. [57], where the Pauli blocking of the occupied electron states is modeled by choosing the discretized mesh with the interval δK in the Brillouin zone. The doping density $n=g_{v}g_{s}/\left(\Omega_{0}N^{2}\right)$ is related to the number of mesh points N×N and the area of the primitive cell $\Omega_{0}$ ($g_{s}$ and $g_{v}$ are spin and valley degeneracies, respectively). The corresponding Fermi energy of the doping electrons is given by $E_{F}=\hbar^{2}\delta k^{2}/2m$ where the discretization interval for the hexagonal lattice of MoS2 is $\delta k=4\pi /(\sqrt{3}aN)$.

## Appendix C. Trion *s_z_τ* = ±3/2 States

The trion states $s^{z}\tau =\pm 3/2$ are fully dark and scattering between $s^{z}\tau =\pm 3/2$ and $s^{z}\tau =\pm 1/2$ requires a nonzero spin-boson. However, in the presence of an intersystem crossing from singlet to triplet excitons, it is possible to obtain the $s^{z}\tau =\pm 3/2$ trion state in the freestanding of the MoS2 monolayer as the lowest trion states, with significant changes in the photoluminescence temperature dependence. The energy splitting between the lowest $s^{z}\tau =\pm 3/2$ state and the lowest $s^{z}\tau =\pm 1/2$ state will be about 2.3 meV in the zero doping level limit, as shown in Figure A1a. This effect is due to the absence of repulsive exchange interaction between the hole and electrons, see Figure A1b, and will also be observed only in the MoS2 monolayer due to the low conduction band splitting, which is comparable to the amplitude of the exchange interaction.
